# The Responsiveness of the Lucerne ICF-Based Multidisciplinary Observation Scale: A Comparison with the Functional Independence Measure and the Barthel Index

**DOI:** 10.3389/fneur.2016.00152

**Published:** 2016-09-26

**Authors:** Tim Vanbellingen, Beatrice Ottiger, Tobias Pflugshaupt, Jan Mehrholz, Stephan Bohlhalter, Tobias Nef, Thomas Nyffeler

**Affiliations:** ^1^Neurology and Neurorehabilitation Center, Luzerner Kantonsspital, Luzern, Switzerland; ^2^Gerontechnology and Rehabilitation Group, University of Bern, Bern, Switzerland; ^3^Wissenschaftliches Institut, Klinik Bavaria in Kreischa GmbH, Kreischa, Germany; ^4^ARTORG Center for Biomedical Engineering Research, University of Bern, Bern, Switzerland

**Keywords:** responsiveness, functional outcome, LIMOS, FIM, BI, stroke

## Abstract

**Background:**

Good responsive functional outcome measures are important to measure change in stroke patients. The aim of study was to compare the internal and external responsiveness, floor and ceiling effects of the motor, cognition, and communication subscales of the Lucerne ICF-based Multidisciplinary Observation Scale (LIMOS) with the motor and cognition subscales of the Functional Independence Measure (FIM), and the Barthel Index (BI), in a large cohort of stroke patients.

**Methods:**

One hundred eighteen stroke patients participated in this study. Admission and discharge score distributions of the LIMOS motor, LIMOS cognition and communication, FIM motor and FIM cognition, and BI were analyzed based on skewness and kurtosis. Floor and ceiling effects of the scales were determined. Internal responsiveness was assessed with *t*-tests, effect sizes (ESs), and standardized response means (SRMs). External responsiveness was investigated with linear regression analyses.

**Results:**

The LIMOS motor and LIMOS cognition and communication subscales were more responsive, expressed by higher ESs (ES = 0.65, SRM = 1.17 and ES = 0.52, SRM = 1.17, respectively) as compared with FIM motor (ES = 0.54, SRM = 0.96) and FIM cognition (ES = 0.41, SRM = 0.88) and the BI (ES = 0.41, SRM = 0.65). The LIMOS subscales showed neither floor nor ceiling effects at admission and discharge (all <15%). In contrast, ceiling effects were found for the FIM motor (16%), FIM cognition (15%) at discharge and the BI at admission (22%) and discharge (43%). LIMOS motor and LIMOS cognition and communication subscales significantly correlated (*p* < 0.0001) with a change in the FIM motor and FIM cognition subscales, suggesting good external responsiveness.

**Conclusion:**

We found that the LIMOS motor and LIMOS cognition and communication, which are ICF-based multidisciplinary standardized observation scales, might have the potential to better detect changes in functional outcome of stroke patients, compared with the FIM motor and FIM cognition and the BI.

## Introduction

Several measures for activities of daily living (ADL) have been published for patients with stroke. Among those, the Barthel index (BI) (1) and the functional independence measure (FIM) (2) are most widely used (3–5). The FIM covers two main aspects of functional outcome, by including a motor and cognitive subscale, while the BI includes motor items only. Previous studies have explored floor and ceiling effects, and responsiveness of both FIM subscales, often comparing the FIM motor subscale with the BI. No clear advantage of the FIM motor subscale over the BI has been found (6, 7). In addition, floor and ceiling effects have been suggested for both FIM motor subscale (7–9) and BI (10, 11). An attempt to overcome ceiling effects and to extend the range of the FIM has been the adding of 12 additional items of the functional assessment measure (FAM) to the FIM, so-called FIM + FAM (12). However, the added value of the FAM can be questioned, since ceiling effects still remained (12, 13). Consequently, the FIM is still most commonly used as a reference functional outcome measurement and this, in particular, in stroke rehabilitation centers (5).

Recently, the Lucerne ICF-based Multidisciplinary Observation Scale (LIMOS) has been developed (14). In this study, it was found that the scale covers four components, which can be defined as LIMOS motor, LIMOS cognition, LIMOS communication, and LIMOS domestic life subscales. These LIMOS subscales have several advantages. First, the composition and rating of the scales are based on the International Classification of Functioning, Disability, and Health (ICF) (15–18). In fact, the selection of the items of the LIMOS is based on the comprehensive ICF core sets for stroke (17). Second, the scales are used by a multidisciplinary team (nurses, physical and occupational therapists, speech therapists, neurologists). Finally, with respect to the LIMOS motor and LIMOS cognition, for example, these include detailed motor items, such as carrying objects (d430), and cognitive items, such as focusing attention (d160). Therefore, the more comprehensive LIMOS subscales are expected to be more sensitive to change over time than the other measures.

The test–retest, inter-rater reliability and construct validity of the total LIMOS and its subscales has been previously confirmed (14). However, the internal and external responsiveness, which are important psychometric properties, still remains to be established. The internal responsiveness is defined as the ability of a measure to change over a specific time frame, and the external responsiveness is reflected by the extent to which changes in a measure relate to corresponding changes in a reference measure (19). The advantage of having more sensitive measures is that even subtle changes can be measured in stroke patients with already good sensory–motor functions. These patients may still have impaired cognitive functions associated with difficulties in extended ADL tasks (e.g., cooking, using public transport services).

The aim of this single center, prospective cohort study was to explore the internal and external responsiveness, floor and ceiling effects, of the LIMOS motor, and LIMOS cognition and communication subscales – relative to the widely used FIM motor and FIM cognition subscales and the BI – in a large cohort of inpatients with stroke, who received multidisciplinary neurorehabilitation.

## Materials and Methods

### Patients

One hundred eighteen inpatients with stroke (37 women; age 41–89 years, mean = 69.4 years, SD = 11.94 years; time interval stroke onset and admission assessment was between 1–64 days, mean = 12.9 days, SD = 9.52 days) were prospectively included. Seventy-nine patients had an ischemic (middle cerebral artery = 50, posterior cerebral artery = 13, anterior cerebral artery = 6, several arterial territories = 10) and 39 patients had a hemorrhagic stroke. All patients were sequentially admitted to the Neurology and Neurorehabilitation Center, Luzerner Kantonsspital (LUKS), from January 2014 to January 2015 and received multidisciplinary neurorehabilitation. The study was approved by the ethical committee of the state of Lucerne and was consistent with the Declaration of Helsinki, 1975. All patients gave informed consent prior to participation.

### Instruments

Each stroke patient was assessed with LIMOS motor, LIMOS cognition and LIMOS communication subscales, and the FIM motor and FIM cognitive subscales at admission and at final discharge (mean duration of length of stay = 28.9 days, SD = 18.9 days). A derived BI score was obtained based on the motor items of the FIM. A previous study demonstrated an almost perfect correlation (rho = 0.99) between an observed and derived BI score (20). The different measurements (LIMOS, FIM, BI) for each single patient were done at admission and discharge by the same assessors.

The previous validation study of the LIMOS revealed that the total scale contains four components, based on a principal component analysis (14). Consequently, the total scale can be divided into a LIMOS motor (including interpersonal activities, mobility, and self-care), LIMOS cognition (including knowledge and general tasks), LIMOS communication, and LIMOS domestic life subscale. For proper comparison with the FIM, the LIMOS motor and LIMOS cognition and communication subscales are of interest in this study. The LIMOS motor subscale contains 20 items, the LIMOS cognitive and communication subscale contain 15 cognitive and 5 communication items. Every item is rated on a 5-point scale (1–5). The 5-point scale for the LIMOS is defined as follows: 1 = patient is not able to fulfill a task or needs assistance up to 75% (corresponding to “complete”); 2 = patient is able to fulfill tasks with assistance of 25 to 75% (corresponding to “severe”); 3 = patient is able to fulfill tasks with assistance less than 25% or under supervision (corresponding to “moderate”); 4 = patient is able to fulfill tasks independently but needs more time and/or with auxiliary materials, aids (corresponding to “slight”); 5 = patient is able to fulfill tasks independently (corresponding to “none”) (for more information regarding item description and assessment of LIMOS, also refer Data Sheet S1 in Supplementary Material).

The FIM is a standardized assessment for ADL, which includes 18 items rated on a 7-point scale: 1 = total assistance; 2 = maximal assistance; 3 = moderate assistance; 4 = minimal contact assistance; 5 = supervision or set-up; 6 = modified independence; and 7 = complete independence (2). The FIM consists of 13 motor (or physical) items and 5 cognitive items. The scores range from 13 to 91 for the motor subscale and from 5 to 35 for the cognitive subscale.

The BI measures self-care and mobility, including 10 items scored at two to four levels (1). According to Collin and colleagues (21), a total score of 20 points can be obtained, which indicate fully independency in ADL.

### Statistical Analyses

In accordance with Husted and colleagues (19), internal responsiveness was examined using three most commonly used statistics being paired *t*-test, the standardized effect size (ES), and the standardized response mean (SRM), which is a different type of ES. The SRM is calculated by dividing the mean difference score by the standard deviation (SD) of the difference score. The ES is calculated as the mean difference score divided by the SD of the baseline score (22, 23). According to Cohen’s criteria, an ES >0.8 is large, 0.5–0.8 is moderate, and 0.2–0.5 is small (24). External responsiveness was investigated by calculating Pearson product moment correlation coefficients (*r*), between changes in LIMOS and FIM subscales. We also performed multiple linear regression analyses of the relationship between the LIMOS and FIM subscales. The LIMOS subscales change score (discharge–admission score) was taken as the dependent variables (*y*), and LIMOS subscales admission scores, and FIM subscale admission scores, and FIM subscale score changes were taken as independent variables.

The score ranges and distributions (skewness and kurtosis) of each of the measures were examined. The floor and ceiling effects reflect the extent to which scores cluster at the bottom and top, respectively, of the scale range. Floor and ceiling effects were considered present if 15% of respondents scored the lowest or highest score on a scale, respectively (23). Score distributions were considered normal if the skewness and kurtosis were between −1 and +1.

Descriptive analyses were applied to patients’ demographic variables and behavioral performances (LIMOS, FIM, BI). Level of significance was set at *p* = 0.05 (two-tailed). All values are expressed as mean ± SD. Statistical analyses were performed using PASW for Windows (version 23.0; SPSS, Inc., Chicago, IL, USA).

## Results

The score distributions of the three measures are presented in detail in Table 1.

**Table 1:** **Score distributions of the LIMOS, FIM, and BI scales**.

	t0	t1	Skewness		Kurtosis		Floor		Ceiling	
	Mean (SD)	Mean (SD)	t0	t1	t0	t1	t0	t1	t0	t1
LIMOS motor	66.70 (22.73)	81.52 (17.51)	−0.41	−1.39	−0.90	1.62	1 (0.8%)	1 (0.8%)	3 (3%)	6 (5%)
LIMOS cog and commun	64.34 (20.81)	74.57 (19.81)	−0.28	−0.70	−0.78	−0.36	1 (0.8%)	0 (0%)	1 (0.8%)	6 (5%)
FIM motor	62.01 (24.29)	75.18 (19.44)	−0.68	−1.61	−0.73	1.78	6 (5%)	1 (0.8%)	5 (4%)	19 (16%)
FIM cog	23.32 (8.44)	26.76 (7.31)	−0.60	−1.02	−0.56	0.53	3 (3%)	1 (0.8%)	7 (6%)	18 (15%)
BI	13.03 (6.48)	15.62 (5.03)	−0.69	−1.74	−0.73	2.23	7 (5.9%)	1 (0.8%)	26 (22%)	51 (43%)

*LIMOS, Lucerne ICF-based multidisciplinary observation scale; FIM, Functional Independence Measure; BI, Barthel Index; t0, measurement at admission; t1, measurement at discharge; cog, cognition; commun, communication; SD, standard deviation*.

With respect to the LIMOS cognition and communication subscale, both admission and discharge scores were normally distributed and not skewed. In contrast, the LIMOS motor, FIM motor, and BI scores were negatively skewed at discharge, indicating that values above the mean are much more frequent. No floor and ceiling effects were found for both LIMOS motor and LIMOS cognition and communication subscales, and this concerned both admission as well as discharge scores. Ceiling effects at discharge were found for the FIM motor and FIM cognition subscales, and the BI. No floor effects were found for these measures.

With regard to the internal responsiveness, highly significant changes over time were found for both LIMOS motor and cognition and communication subscales and also for the FIM motor and cognition subscales as well as the BI. With respect to the magnitude of these changes, both standardized ES and SRM’s were largest for the LIMOS motor and LIMOS cognition and communication (for details see Table 2).

**Table 2:** **Measures of internal responsiveness (mean score difference, effect size, standardized response mean) for the LIMOS motor, LIMOS cognition and communication, FIM motor and cognition and BI**.

Measures	t1–t0 Diff (SD)	*p*-value	Effect size	SRM
LIMOS motor	14.81 (12.69)	<0.0001	0.65	1.17
LIMOS cog and commun	10.23 (8.73)	<0.0001	0.52	1.17
FIM motor	13.17 (13.75)	<0.0001	0.54	0.96
FIM cognition	3.44 (3.90)	<0.0001	0.41	0.88
Barthel	2.65 (4.06)	<0.0001	0.41	0.65

*LIMOS, Lucerne ICF-based multidisciplinary observation scale; FIM, Functional Independence Measure; cog, cognition; commun, communication; SD, standard deviation*.

**Figure 1:**
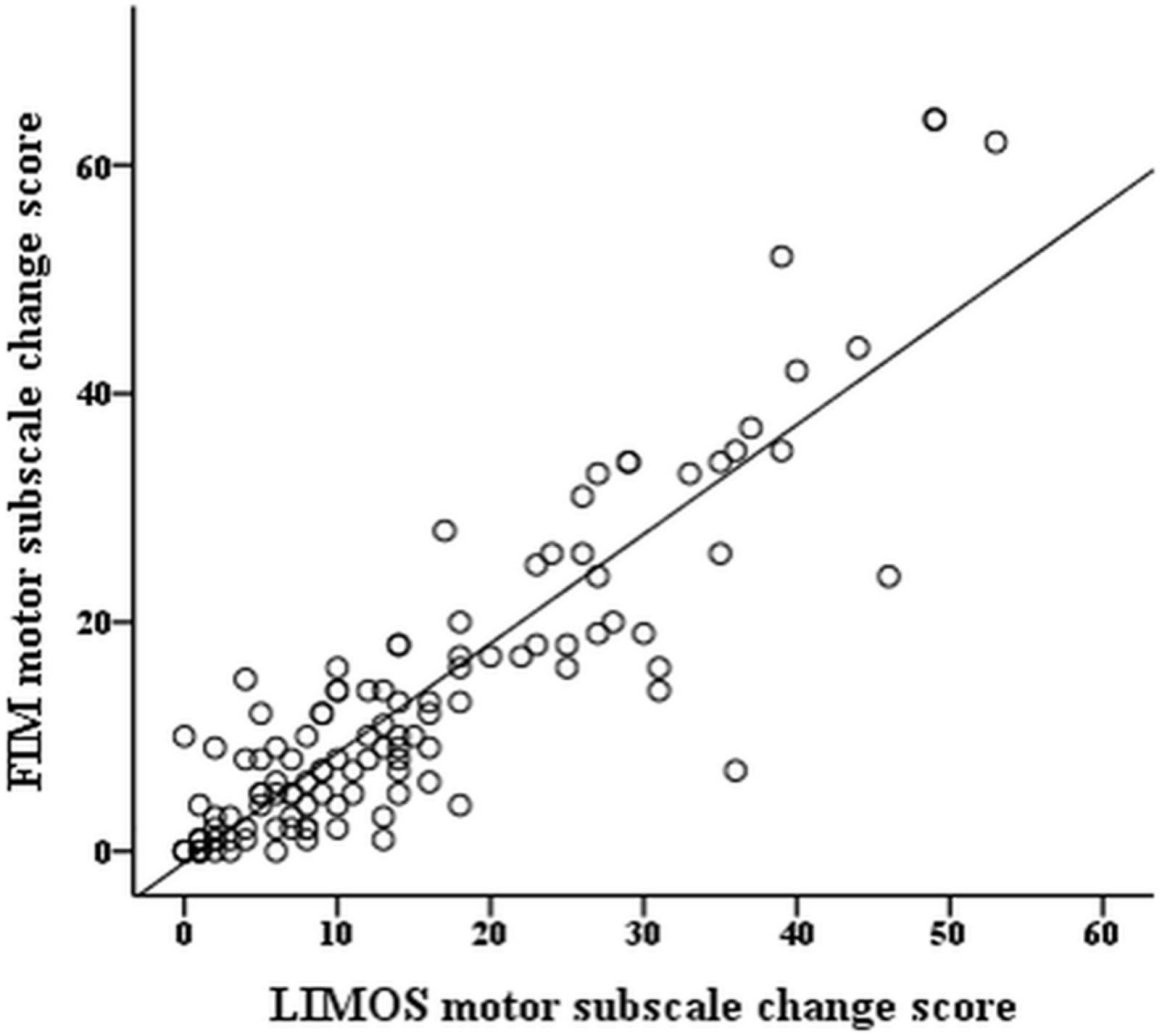
**LIMOS motor subscale change correlates significantly with a change in the FIM motor subscale**.

**Figure 2:**
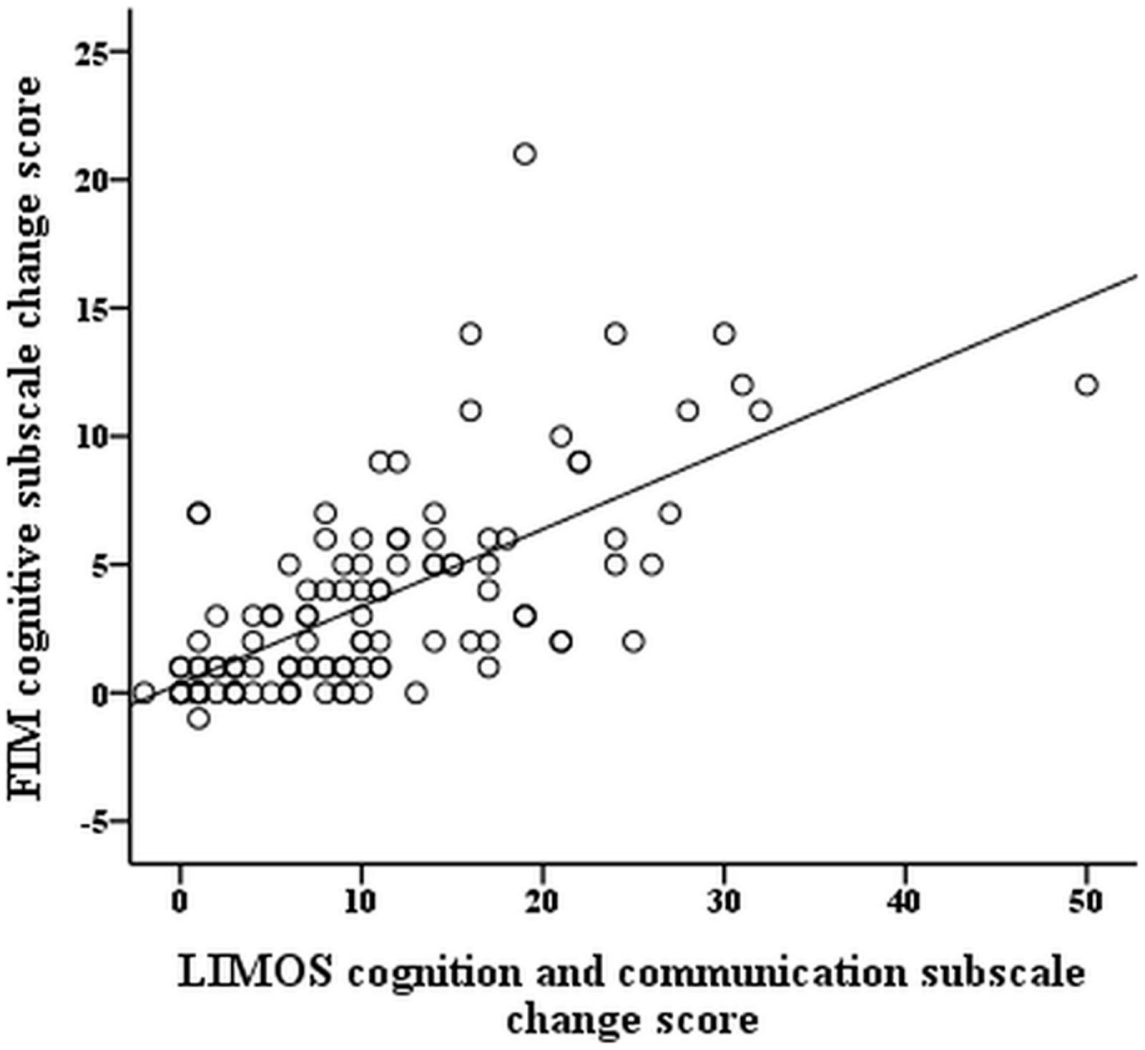
**LIMOS cognition and communication subscale change correlates significantly with a change in the FIM cognition subscale**.

The *r*-values of the Pearson product moment correlation analyses indicate that both LIMOS motor and LIMOS cognition and communication subscales were significantly correlated with a change in the FIM motor and FIM cognition subscales, suggesting good external responsiveness (see also Table 3, and Figures 1 and 2). The multiple linear regression analyses also demonstrated strong significant beta regression coefficients for both LIMOS motor and LIMOS cognition and communication subscales, showing the magnitude of change in both subscales associated with a change in the FIM subscales. The amount of variation in the LIMOS motor subscale change score (improvement from baseline) explained by the FIM motor change score was 84%. For the LIMOS cognition and communication subscales change the amount of variation explained by the FIM cognition change score was 49%.

**Table 3:** **Measures of external responsiveness (correlation method and linear regression analysis) for the LIMOS motor and LIMOS cognition and communication with FIM motor and cognition**.

Measures	*r*	*p*-value	β (SE)	*p*-value	*R*^2^
LIMOS motor	0.88	<0.0001	0.73 (0.04)	<0.0001	0.84
LIMOS cog and commun	0.67	<0.0001	1.63 (0.18)	<0.0001	0.49

*LIMOS, Lucerne ICF based multidisciplinary observation scale; FIM, Functional Independence Measure; SE, standard error; cog, cognition; commun, communication*.

*r, correlation coefficient between the LIMOS subscale change score and the FIM subscale change score*.

*The Beta coefficients stand for the FIM change scores*.

*R^2^ = expresses the regression model statistics of the association between the LIMOS and FIM subscales*.

## Discussion

The main finding of the present study is that the motor, cognition and communication subscales of the LIMOS – which is an ICF-based, comprehensive, reliable and valid assessment measuring functional outcome in stroke patients (14) – are more responsive than the motor and cognition subscales of the FIM, and the BI. Our data further demonstrate that the LIMOS subscales are less prone to ceiling effects than the FIM and the BI. A major advantage of the LIMOS is, therefore, that changes in functional ability can be detected in stroke patients with better baseline levels of motor and cognitive functions at rehabilitation admission, and both this at a motor and a cognitive level.

The good responsiveness of the LIMOS motor and LIMOS cognition and communication subscales may be explained by the comprehensiveness. On the one hand, several cognitive aspects, such as thinking (d163), or solving complex problems (d1751), are integrated in the LIMOS subscale, but not in the FIM cognition subscale. On the other hand, different aspects of mobility (e.g., lifting and carrying objects) are assessed within the LIMOS motor subscale, relative to FIM and BI. This might also explain why the latter two demonstrate ceiling effects, which have already been previously described (6, 7, 10, 11), and are further supported by our data. Ceiling effects clearly limit the usefulness of a tool in assessing change during rehabilitation (25). Interestingly, the importance of integrating more detailed cognitive functions in scales, which measure functional outcome, has already been suggested more than two decades ago (26). In this study, it was shown that deficits in cognition, particularly cognitive abilities, such as short-term verbal memory or comprehension deficits, negatively influence length of stay and are highly predictive for the functional status of a stroke patient at the end of the hospital stay. Even patients who improve physically may not show comparable improvement in cognition and, therefore, may leave rehabilitation with a lesser degree of functional independency and an increased need for follow-up services (26). Consequently, the authors already pledged for an early, comprehensive assessment of deficits in cognition, which may affect a stroke patient’s functional outcome. More recently, two studies further pinpointed to the predictive value of disturbed cognitive functioning in stroke (27, 28).

With respect to the FIM and the BI, our ES and SRM values are similar to those previously found by Wallace and colleagues (29) in stroke patients. Hobart and colleagues (6) found lower responsiveness values for the FIM motor subscale (=0.48) and the BI (=0.56). These different values suggest that the responsiveness of an instrument is strongly related to the timing of the measurements and phase of rehabilitation. Indeed, the two assessment time points (discharge assessment at the latest 3 months after stroke) in our cohort were similar to those of Wallace and colleagues’ (29) study, explaining the similar results, whereas Hobart and colleagues (6) followed up patients for a longer time period. In any case, irrespective of the SRM values, we found, for the FIM motor and FIM cognition subscales and BI, much higher ES and SRM values were found for the LIMOS motor and LIMOS cognition and communication subscales. Regarding FIM, an expansion of the scale has been proposed by adding the FAM, which includes additional items for cognition and speech. However, the responsiveness of the expanded FIM (=FIM + FAM) did not further improve (12), which might be explained by the fact that these additional items were not specific enough to detect any further change (6). With the LIMOS cognition and communication, this problem could now be overcome.

One could argue that due to the more comprehensive nature of the LIMOS, its administration might be rather time-consuming, and, indeed, the total number of LIMOS items is higher than those of the FIM or BI. However, it is proportionally lower for each discipline involved (rehabilitation nurse, occupational-, physical-, and speech therapist, or neurologist). The multidisciplinary use of the LIMOS is a major advantage compared with the FIM or BI, which are mostly assessed by nurses only. Since multidisciplinary team care in a neurorehabilitation setting leads to best functional outcome in stroke patients (30), a multidisciplinary assessment should be mandatory.

To conclude, this study demonstrates that the motor and cognition and communication subscales of the LIMOS are highly responsive in stroke. Due to its sensitive properties, the scales have a strong potential to become standard tools in future large randomized controlled rehabilitation trials.

## Author Contributions

Study design: TV, BO, TP, JM, ToN, SB, and ThN. Data acquisition: TV, BO, and TP. Data analysis: TV, BO, and JM. Interpretation of data: TV, BO, TP, JM, ToN, SB, and ThN. Drafting and revising: TV, BO, TP, JM, ToN, SB, and ThN. Final approval: TV, BO, TP, JM, ToN, SB, and ThN.

## Conflict of Interest Statement

The authors declare that the research was conducted in the absence of any commercial or financial relationships that could be construed as a potential conflict of interest.

## References

[1] Mahoney FI Barthel DW Functional evaluation: the Barthel Index. Md State Med J (1965) 14:61–5. 14258950

[2] Keith RA Granger CV Hamilton BB Sherwin FS The functional independence measure: a new tool for rehabilitation. Adv Clin Rehabil (1987) 1:6–18. 3503663

[3] Dromerick AW Edwards DF Diringer MN. Sensitivity to changes in disability after stroke: a comparison of four scales useful in clinical trials. J Rehabil Res Dev (2003) 40(1):1–8. 10.1682/JRRD.2003.01.0001 15150715

[4] Kwon S Hartzema AG Duncan PW Min-Lai S. Disability measures in stroke: relationship among the Barthel Index, the Functional Independence Measure, and the Modified Rankin Scale. Stroke (2004) 35:918–23. 10.1161/01.STR.0000119385.56094.32 14976324

[5] Harvey RL. Predictors of functional outcome following stroke. Phys Med Rehabil Clin N Am (2015) 26(4):583–98. 10.1016/j.pmr.2015.07.002 26522899

[6] Hobart JC Lamping DL Freeman JA Langdon DW McLellan DL Greenwood RJ Evidence-based measurement: which disability scale for neurologic rehabilitation? Neurology (2001) 57:639–44. 10.1212/WNL.57.4.639 11524472

[7] Hsueh IP Lin JH Jeng JS Hsieh CL. Comparison of the psychometric characteristics of the functional independence measure, 5 item Barthel index, and 10 item Barthel index in patients with stroke. J Neurol Neurosurg Psychiatry (2002) 73(2):188–90. 10.1136/jnnp.73.2.188 12122181 PMC1737984

[8] Brock KA Goldie PA Greenwood KM. Evaluating the effectiveness of stroke rehabilitation: choosing a discriminative measure. Arch Phys Med Rehabil (2002) 83:92–9. 10.1053/apmr.2002.27348 11782838

[9] Coster WJ Haley SM Jette AM. Measuring patient-reported outcomes after discharge from inpatient rehabilitation settings. J Rehabil Med (2006) 38(4):237–42. 10.1080/16501970600609774 16801206

[10] Duncan PW Samsa GP Weinberger M Goldstein LB Bonito A Witter DM Health status of individuals with mild stroke. Stroke (1997) 28(4):740–5. 10.1161/01.STR.28.4.740 9099189

[11] Hsueh IP Lee MM Hsieh CL. Psychometric characteristics of the Barthel activities of daily living index in stroke patients. J Formos Med Assoc (2001) 100(8):526–32. 11678002

[12] Linn RT Blair RS Granger CV Harper DW O’Hara PA Maciura E. Does the functional assessment measure (FAM) extend the functional independence measure (FIM) instrument? A rasch analysis of stroke inpatients. J Outcome Meas (1999) 3(4):339–59. 10572386

[13] Turner-Stokes L Siegert RJ. A comprehensive psychometric evaluation of the UK FIM+FAM. Disabil Rehabil (2013) 35(22):1885–95. 10.3109/09638288.2013.766271 23384240 PMC3812697

[14] Ottiger B Vanbellingen T Gabriel C Huberle E Koenig-Bruhin M Pflugshaupt T Validation of the new Lucerne ICF based Multidisciplinary Observation Scale (LIMOS) for stroke patients. PLoS One (2015) 10(6):e0130925 10.1371/journal.pone.0130925 26110769 PMC4481343

[15] WHO. International Classification of Functioning, Disability and Health: ICF. Geneva: World Health Organization (2001).

[16] Cieza A Ewert T Üstün B Chatterji S Kostanjsek N Stucki G. Development of ICF core SETS for patients with chronic conditions. J Rehabil Med (2004) 36:9–11. 10.1080/16501960410016046 15370742

[17] Geyh S Cieza A Schouten J Dickson H Frommelt P Omar Z ICF core sets for stroke. J Rehabil Med (2004) 44(Suppl):135–41. 10.1080/16501960410016776 15370761

[18] Stucki G Grimby G Foreword: applying the ICF in medicine. J Rehabil Med (2004) 36:5–6. 10.1080/16501960410022300 15370740

[19] Husted JA Cook RJ Farewell VT Gladman DD. Methods for assessing responsiveness: a critical review and recommendations. J Clin Epidemiol (2000) 53(5):459–68. 10.1016/S0895-4356(99)00206-1 10812317

[20] Nyein K McMichael L Turner-Stokes L. Can a Barthel score be derived from the FIM? Clin Rehabil (1999) 13:56–63. 10.1191/026921599701532135 10327098

[21] Collin C Wade DT Davies S Horne V. The Barthel ADL Index: a reliability study. Int Disabil Stud (1988) 10(2):61–3. 10.3109/09638288809164103 3403500

[22] Kazis LE Anderson JJ Meenan RF. Effect sizes for interpreting changes in health status. Med Care (1989) 27:S178–89. 10.1097/00005650-198903001-00015 2646488

[23] Terwee CB Bot SD de Boer MR van der Windt DA Knol DL Dekker J Quality criteria were proposed for measurement properties of health status questionnaires. J Clin Epidemiol (2007) 60:34–42. 10.1016/j.jclinepi.2006.03.012 17161752

[24] Cohen J Statistical Power Analysis for the Behavioral Sciences. 2nd ed Hillsdale, NJ: Erlbaum (1988).

[25] Stineman MG Shea JA Jette A Tassoni CJ Ottenbacher KJ Fiedler R The Functional Independence Measure: tests of scaling assumptions, structure, and reliability across 20 diverse impairment categories. Arch Phys Med Rehabil (1996) 77(11):1101–8. 10.1016/S0003-9993(96)90130-6 8931518

[26] Galski T Bruno RL Zorowitz R Walker J. Predicting length of stay, functional outcome, and aftercare in the rehabilitation of stroke patients. The dominant role of higher-order cognition. Stroke (1993) 24(12):1794–800. 10.1161/01.STR.24.12.1794 8248957

[27] van der Zwaluw CS Valentijn SA Nieuwenhuis-Mark R Rasquin SM van Heugten CM. Cognitive functioning in the acute phase poststroke: a predictor of discharge destination? J Stroke Cerebrovasc Dis (2011) 20(6):549–55. 10.1016/j.jstrokecerebrovasdis.2010.03.009 20833083

[28] Park YH Jang JW Park SY Wang MJ Lim JS Baek MJ Executive function as a strong predictor of recovery from disability in patients with acute stroke: a preliminary study. J Stroke Cerebrovasc Dis (2015) 24(3):554–61. 10.1016/j.jstrokecerebrovasdis.2014.09.033 25534371

[29] Wallace D Duncan PW Lai SM. Comparison of the responsiveness of the Barthel Index and the motor component of the Functional Independence Measure in stroke: the impact of using different methods for measuring responsiveness. J Clin Epidemiol (2002) 55(9):922–8. 10.1016/S0895-4356(02)00410-9 12393081

[30] Momsen AM Rasmussen JO Nielsen CV Iversen MD Lund H. Multidisciplinary team care in rehabilitation: an overview of reviews. J Rehabil Med (2012) 44(11):901–12. 10.2340/16501977-1040 23026978

